# Cell growth inhibition and apoptotic effects of a specific anti-RTFscFv antibody on prostate cancer, but not glioblastoma, cells

**DOI:** 10.12688/f1000research.10803.1

**Published:** 2017-02-17

**Authors:** Foroogh Nejatollahi, Payam Bayat, Bahareh Moazen

**Affiliations:** 1 Shiraz HIV/AIDS research center, Institute of Health, Shiraz University of Medical Sciences, Shiraz, Iran; 2 Recombinant Antibody Laboratory, Department of Immunology, Shiraz University of Medical Sciences, Shiraz, Iran

**Keywords:** Prostate cancer, Anti-RTF scFv, Growth inhibition, Apoptosis, Immunotherapy

## Abstract

**Background: **Single chain antibody (scFv) has shown interesting results in cancer immunotargeting approaches, due to its advantages over monoclonal antibodies. Regeneration and tolerance factor (RTF) is one of the most important regulators of extracellular and intracellular pH in eukaryotic cells. In this study, the inhibitory effects of a specific anti-RTF scFv were investigated and compared between three types of prostate cancer and two types of glioblastoma cells. **Methods: **A phage antibody display library of scFv was used to select specific scFvs against RTF using panning process. The reactivity of a selected scFv was assessed by phage ELISA. The anti-proliferative and apoptotic effects of the antibody on prostate cancer (PC-3, Du-145 and LNCaP) and glioblastoma (U-87 MG and A-172) cell lines were investigated by MTT and Annexin V/PI assays. **Results: **A specific scFv with frequency 35% was selected against RTF epitope. This significantly inhibited the proliferation of the prostate cells after 24 h. The percentages of cell viability (using 1000 scFv/cell) were 52, 61 and 73% for PC-3, Du-145 and LNCaP cells, respectively, compared to untreated cells. The antibody (1000 scFv/cell) induced apoptosis at 50, 40 and 25% in PC-3, Du-145 and LNCaP cells, respectively. No growth inhibition and apoptotic induction was detected for U-87 and A172 glioblastoma cells. **Conclusions: **Anti-RTFscFv significantly reduced the proliferation of the prostate cancer cells. The inhibition of cell growth and apoptotic induction effects in PC-3 cells were greater than Du-145 and LNCaP cells. This might be due to higher expression of RTF antigen in PC-3 cells and/or better accessibility of RTF to scFv antibody. The resistance of glioblastoma cells to anti-RTF scFv offers the existence of mechanism(s) that abrogate the inhibitory effect(s) of the antibody to RTF. The results suggest that the selected anti-RTF scFv antibody could be an effective new alternative for prostate cancer immunotherapy.

## Introduction

Prostate cancer is the most prevalent malignancy and the second leading cause of cancer-related death among men in the USA and developing countries ^1^. Several new strategies have been employed to manage prostate cancer, including gene therapy, targeted therapy with prodrugs, angiogenesis inhibition and immunotherapy ^2, 3^. In order to exploit the immune system to retard or even stop tumor cell growth, either via targeting tumor antigens or by disturbing signaling pathways, immunotherapy is a very beneficial method that has been developed ^4^. In recent years, monoclonal antibody-based immunotherapy has been used to target prostate-associated antigens ^5, 6^. Targeting prostate-associated antigens may make conventional therapeutic regimens, including chemotherapy and radiotherapy, more beneficial if applied in combination ^7^. To provide an effective targeted therapy, a number of prostate cancer-related antigens have been used, including prostate-specific antigen (PSA), prostate specific membrane antigen (PSMA), prostatic acid phosphatase, Prostatic stem cell antigen (PSCA) and kalikrein-4 (KLK4) ^8– 12^. Regeneration and tolerance factor (RTF), a novel membrane protein, has also been introduced as a new attractive target for immunotherapy, since its overexpression has been observed in many kinds of malignant and metastatic cancers, and it has been shown to exert immunoregulatory properties ^13, 14^. RTF is the a _2_ isoform of V _0_ subunit, which is one of the vacuolar H ^+^-ATPase (V-ATPase) proton pumps and participates in the control of pH in normal and tumor cells via proton pumping across the membrane to the extracellular space or intracellular organelles, which, in turn, contributes to extracellular acidification and maintenance of relatively neutral cytosolic pH ^15^. Acidifying the tumor microenvironment plays a key role in tumor cell proliferation, metastasis and resistance to chemotherapy ^13, 14^. It has been shown that anti-RTF monoclonal antibody can block RTF-ATPase activity and induces apoptosis in a Jurkat T cell line expressing RTF ^16^. Bermudez *et al*. ^17^ have demonstrated that the RTF molecule is expressed in highly metastatic prostate cancer cells and inhibiting V-ATPase enhances chemosensitivity in metastatic prostate cancer.

Recombinant DNA technology paved the way for the production of recombinant antibody (rAb) fragments, such as single-chain variable fragment (scFv) antibodies, which are composed of variable heavy (V _H_) and light (V _L_) chains linked by a flexible peptide linker ^18– 21^. Properties of scFv antibodies, including smaller molecular size, human origin and better penetration to the target compared with whole antibodies, make these molecules suitable for therapeutic applications ^22– 24^. In the present study, the inhibitory effects of selected anti-RTF scFvs on three prostate cancer cell lines, PC-3, Du-145 and LNCaP cells, and two glioblastoma cell lines, U-87 MG and A-172, were investigated.

## Methods

### Selection of anti-RTF scFv antibody

A phage antibody display library of scFv was developed as described previously ^18, 19^. Briefly, panning process was performed to enrich the phage library. The RTF peptide amino acids 488–510 was employed as the target antigen. The peptide was diluted (10μg/ml) and coated in a polystyrene immunotube (Nunc, Finland). After an overnight incubation, washing was performed with PBS and blocking solution (10% FCS [Sigma, UK] and 2% skimmed milk in PBS) was added to the tube and was incubated at 37°C for 2 h. After washing four times with PBS/Tween (PBST) and four times with PBS, phage supernatant diluted with blocking solution (1:1) was added and incubated at room temperature for 1 h. The tube was washed, logarithmic phase TG1 *E. coli* (Sigma, UK) ** was added and incubated at 37°C for 1 h. The pellet was obtained with centrifugation at 3000 rpm for 5 min, resuspended in 200 μl of 2TY broth and plated onto 2TYG Agar/Ampicilin plate and incubated at 30°C overnight. Panning process was performed for four rounds to obtain specific scFv antibodies against the desired peptide. The V _H_-Linker-V _L_ inserts of selected scFv clones were PCR amplified (denaturation 1 min, annealing 1 min, elongation 2 min; R1 and R2 vector primers). Mva1 fingerprinting (Sigma, UK) was performed on 20 colonies of the panned library to determine the homogenicity and frequency of positive samples of PCR products.

### Phage ELISA

The RTF peptide was diluted to 100μg/ml and coated in 96 wells polystyrene plate (Nunc, Denmark). The plate was incubated at 4°C overnight. The wells containing no peptide, unrelated peptide, M13KO7 helper phage (New England Biolabs, UK) and unrelated scFv (scFv against HER2 ^21^) were also considered as controls. All the wells were in triplicate. The wells were washed three times with PBST and three times with PBS. A 150μl of 2% skimmed milk were added to each well as blocking solution, and incubation was performed at 37°C for 2h. The wells were washed and diluted phage (10 ^9^ PFU/ml) was added to each well. M13KO7 was also added to the wells allocated for helper phage instead of phage antibody. The plate was incubated at room temperature for 2h. Nonbinding phages were removed by washing with PBST and PBS, and diluted anti-Fd rabbit antibody (1/100; catalog no., B7786; Sigma, UK) ^19^ was added to each well and incubated at room temperature for 1.5h. Following washing, peroxidase conjugated goat anti-rabbit IgG (1/4000; catalog no., A0545; Sigma) ^19^ was added to each well and incubated at room temperature for 1h. Nonbinding antibodies were removed by washing and 0.5 mg/ml of ABTS (Sigma, USA) in citrate buffer/H _2_O _2_ was added. The optical density of each well was read at 405 nm.

### Cell culture

Human prostate cancer cell lines, PC-3, Du-145 and LNCaP, and human glioblastoma cell lines, U-87 MGand A-172, were purchased from National Cell Bank of Iran, Pasteur Institute of Iran (Tehran, Iran). The cells were cultured and maintained in RPMI 1640 (Biosera, UK) in CO _2_ incubator at 37°C. The medium was supplemented with 10% FBS (Biosera, UK), 100U/ml penicillin and 100 μg/ml streptomycin.

### Cell proliferation assay

Each cell line was transferred into a 96-well flat-bottomed plate (10 ^4^ cells per well) and incubated at 37°C overnight. The cells were treated in triplicate with different concentrations of anti-RTF scFv antibodies (100, 200, 500, 1000 scFv/cell); M13KO7 and 2TY broth media were used as negative controls. After a 24h treatment at 37°C, MTT [3-(4, 5-dimethylthiazol-2, 5-diphenyltetrazolium bromide, 0.5 mg/ml; Sigma, Germany] was added to each well and incubated at 37°C for 4 hrs. The supernatant was removed and the crystal products were dissolved by adding DMSO (Merck, Germany) and incubation at room temperature overnight. Colorimetric evaluation was performed at 490 nm. The percentage of cell growth was calculated from the absorbance value of untreated and treated cells as follows: percentage of cell growth = (OD _490_ treated / OD _490_ untreated) × 100.

### Annexin V-FITC assay

Capability of the selected scFv in inducing apoptosis in the prostate and glioblastoma cells were investigated by Annexin-V/propidium iodide (PI) assay. In total, 8×10 ^5^cells were seeded per culture plate and incubated overnight at 37°C. The cells were treated with anti-RTF scFv antibody (1000 scFv/cell) for 24 h. Untreated cells were considered as negative control. The cells were harvested using 0.25% trypsin/EDTA, washed with cold PBS and transferred into flow cytometry tubes followed by adding Annexin V-FITC and PI to the both treated and untreated cells. Preparation was completed by adding incubation buffer (Roche Applied Science, Germany) to each tube. The tubes belonged to the 5 cell lines were read with BD FACSCalibur (Becton Dickinson, Franklin Lakes, NJ, USA) and analyzed by WinMDI 2.5 software.

### Statistical analysis

The data obtained from cell proliferation assays were statistically analyzed by ANOVA test using GraphPad Prism 5 software to compare the means of percentages of cell growth between treated and untreated cells. All data are presented as means ± standard deviation (SD). *p value*<0.05 was considered statistically significant.

## Results

### Selection of anti-RTF scFv antibody

**Figure 1.**
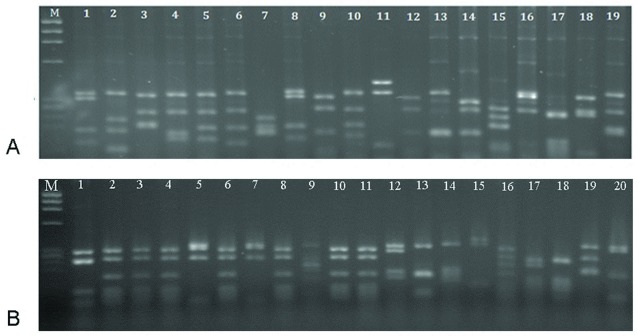
DNA fingerprinting of scFv clones. ( **A**) Heterogeous patterns were obtained for the un-panned library. A common pattern with frequency 35% (lanes 2, 3, 4, 6, 8, 10 and 11) demonstrated the selection of specific scFv after panning. ( **B**) Marker – øX174 DNA (72–1353 bp).

DNA fingerprinting of the library clones and the selected clones obtained after four rounds of panning are shown in Figure 1. The different patterns of the library clones demonstrated a diverse and heterogeous library. After panning, a predominant pattern with frequency 35% (lanes 2, 3, 4, 6, 8, 10, and 11) was obtained, which was considered as selected scFv against RTF for following experiments.

### Phage ELISA

**Figure 2.**
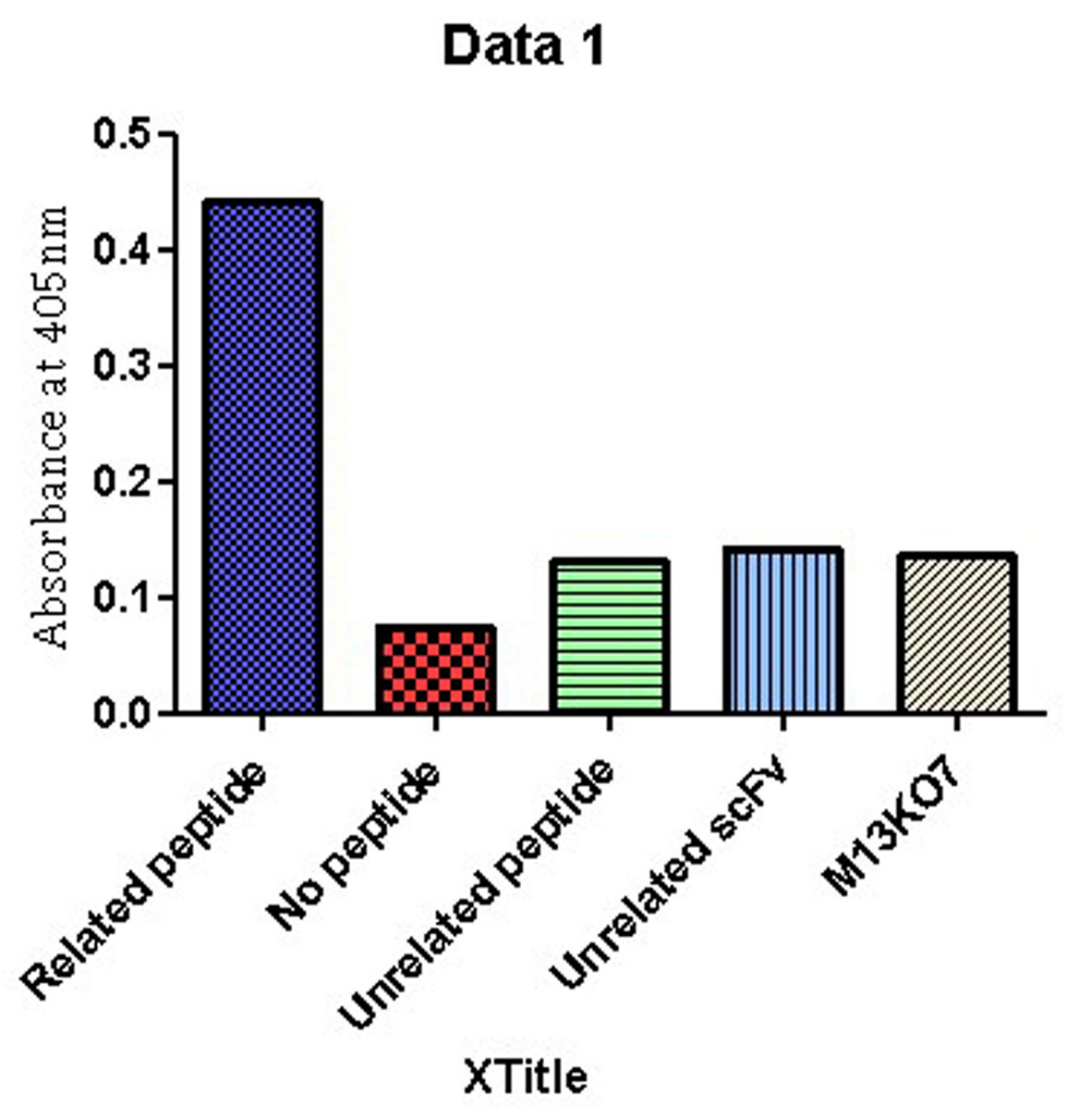
Phage ELISA result of the selected clone. Plates were set in duplicates and wells in tetraplicates.

To evaluate the reactivity of the scFv antibody to the RTF peptide, phage ELISA was performed. The anti-RTF scFv antibodies produced positive ELISA and the average OD was 0.441 at 405 nm ( Figure 2). The baseline reading from the wells with no peptide was 0.075. Unrelated peptide, unrelated scFv and M13KO7 wells showed an average absorbance of 0.132, 0.142, and 0.136, respectively.

Phage ELISA raw data Click here for additional data file. Copyright: © 2017 Nejatollahi F et al. 2017 Data associated with the article are available under the terms of the Creative Commons Zero "No rights reserved" data waiver (CC0 1.0 Public domain dedication).

### Cell proliferation assay

**Figure 3.**
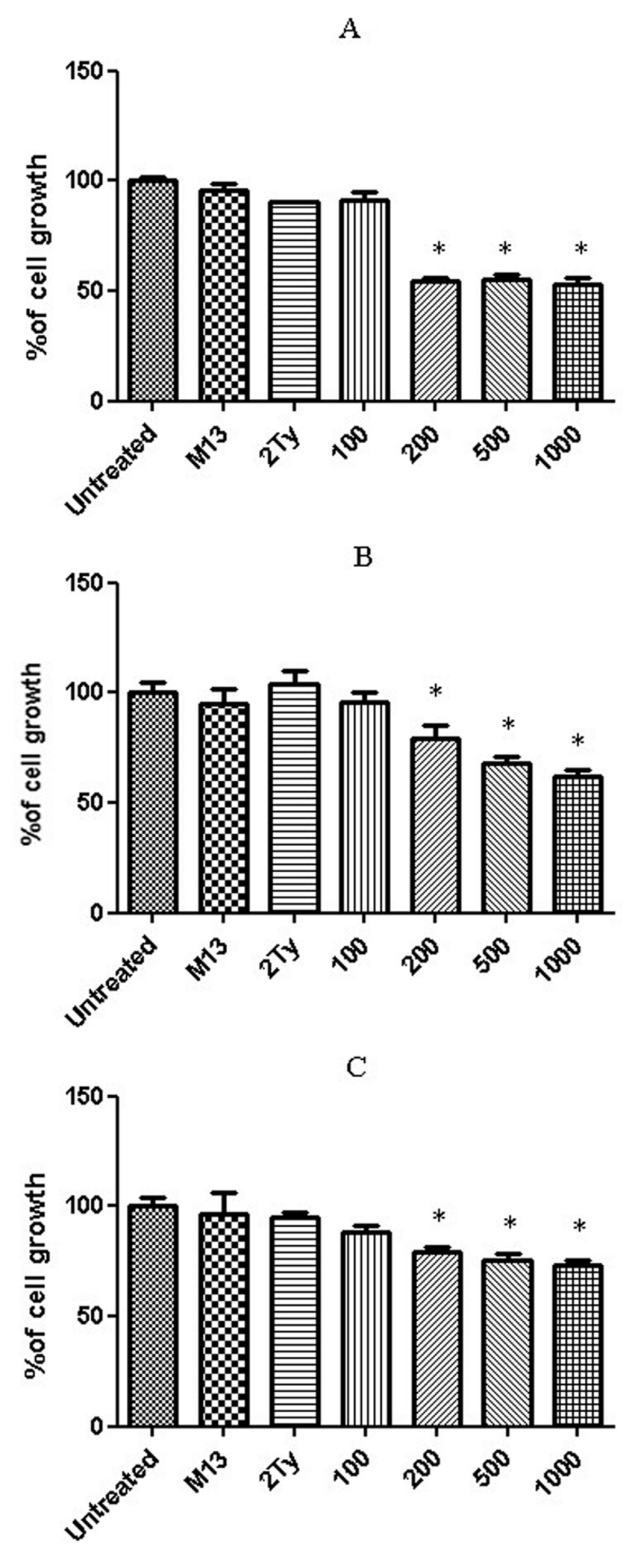
Percentage of prostate cell growth after treatment with anti-RTF scFv. Growth percentage of ( **A**) PC-3, ( **B**) DU-145 and ( **C**) LNCaP cell lines after 24 h treatment with 100, 200, 500 and 1000 anti-RTF scFv/cell. Results of six experiments; * *P value*< 0.05.

**Figure 4.**
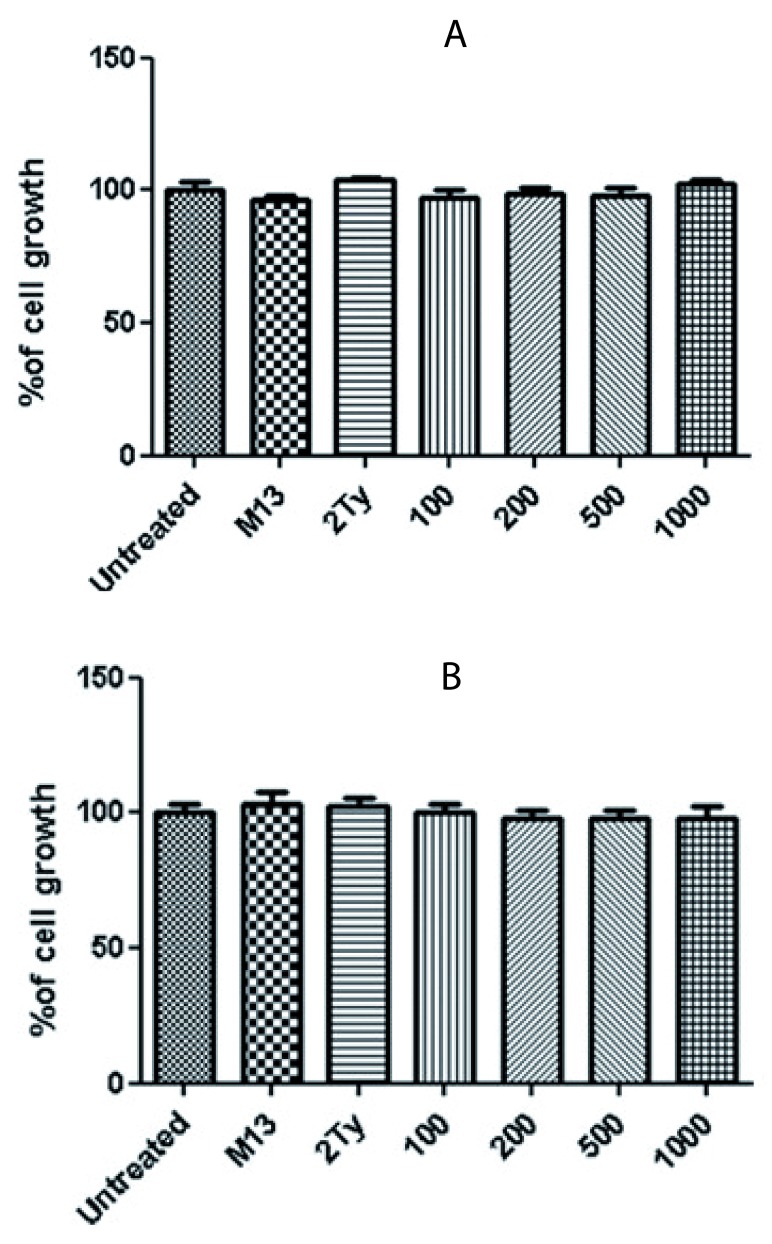
Percentage of glioblastoma cell growth after treatment with anti-RTF scFv. Growth percentage of ( **A**) U-87 and ( **B**) A-172 cell lines after 24 h treatment with 100, 200, 500 and 1000 anti-RTF scFv/cell. Non-significant growth reduction was observed. Results of six experiments; * *P value*< 0.05.

The percentage of cell viability after a 24h treatment with anti-RTF scFv for prostate cancer cell lines are shown in Figure 3. Three concentrations 200, 500 and 1000 scFv/cell demonstrated significant cell inhibition growth in the three cell lines ( *P value* < 0.05). The best growth inhibition was at a concentration of 1000 scFv/cell, and the percentage of cell growth for PC-3, DU-145 and LNCaP cells at these concentrations were 52, 61 and 73%, respectively. No inhibitory effect was observed when the cells were treated with M13KO7 helper phage and 2TY media (negative controls). No significant growth inhibition was detected for glioblastoma cell lines, U-87 MG and A-172 ( Figure 4).

Cell proliferation assay (MTT assay) raw data of three prostate cancer and two glioblastoma cell lines Click here for additional data file. Copyright: © 2017 Nejatollahi F et al. 2017 Data associated with the article are available under the terms of the Creative Commons Zero "No rights reserved" data waiver (CC0 1.0 Public domain dedication).

### Apoptosis effects of anti-RTF scFv

**Figure 5.**
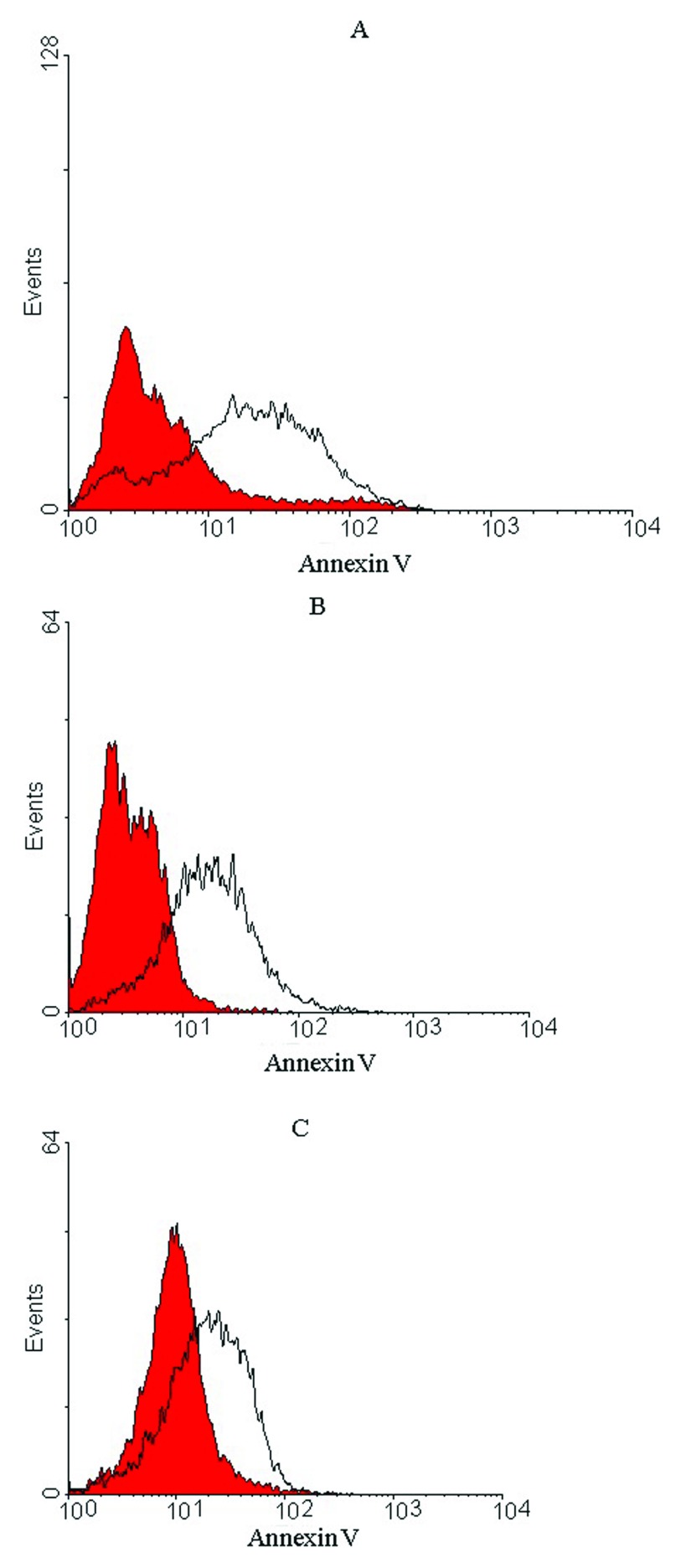
Histograms of untreated and treated prostate cancer cells after 24h treatment with anti-RTF scFv/cell. Representative histograms of untreated cells (red) and treated cells (outlined by a black line) after 24 h incubation period for ( **A**) PC-3, ( **B**) DU-145 and ( **C**) LNCaP prostate cells. A shift in fluorescence intensity observed for the treated cells demonstrated apoptotic cells. Apoptosis occurred in 50% of PC-3, 40% of DU-145 and 25% of LNCaP cells compared to untreated cells

**Figure 6.**
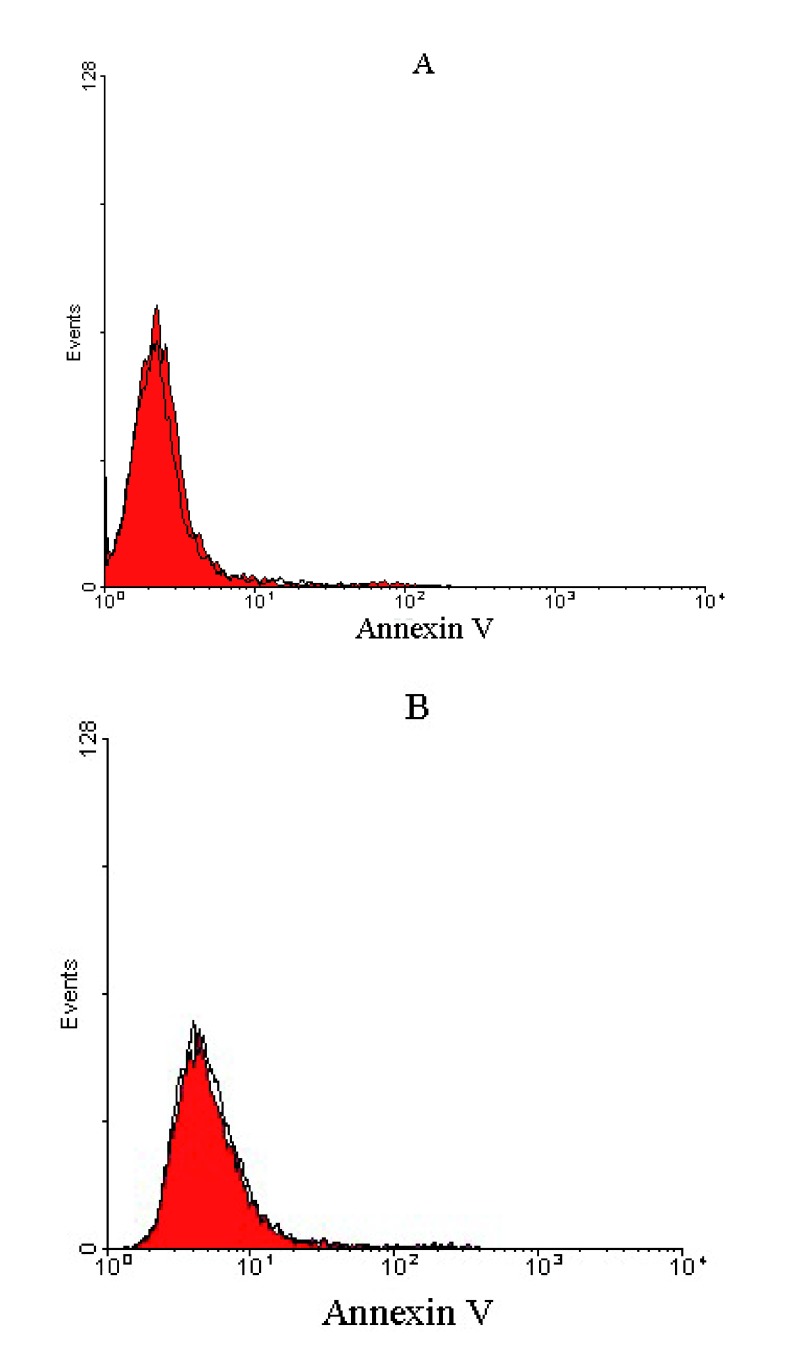
Histograms of untreated and treated glioblastoma cells after 24h treatment with anti-RTF scFv/cell. Representative histograms of untreated cells (red) and treated cells (outlined by a black line) after 24 h incubation period for ( **A**) U-87 MG and ( **B**) A-172 glioblastoma cells. Untreated and treated glioblastoma cells overlapped, representing that the treated cells were viable and non-apoptotic.

Apoptosis was induced in prostate cancer cell lines after a 24 h treatment with 1000 scFv/cell. In total, 50, 40 and 25% of PC-3, Du-145 and LNCaP prostate cancer cells, respectively, showed apoptotic cell death ( Figure 5), whereas no apoptosis was detected for U-87 MG and A-172 glioblastoma cell lines, representing that the treated cells were viable ( Figure 6).

Apoptosis raw data for three prostate cancer and two glioblastoma cell lines Click here for additional data file. Copyright: © 2017 Nejatollahi F et al. 2017 Data associated with the article are available under the terms of the Creative Commons Zero "No rights reserved" data waiver (CC0 1.0 Public domain dedication).

## Discussion

Recombination DNA technology enables the production of human scFv fragments with desirable properties for tissue penetration; therefore, providing immunotherapeutic reagents for targeted therapy of cancers ^25, 26^. The potential role of scFvs in targeted therapy of melanoma, lung, breast, colorectal and prostate cancers have been shown previously ^25, 27– 30^). To isolate a functional scFv, an identified cell target should be selected ^31^. Due to RTF function, which regulates pH in tumor milieu, it has been considered as an ideal target for cancer immunotherapy, and an anti-RTF monoclonal antibody has been capable of inducing apoptosis in an ovarian carcinoma cell line ^13^.

In the present study, we applied scFv antibodies to target the RTF molecule in both prostate and glioblastoma cancer cells. Amino acids 488–510 of RTF, which was used to isolate anti-RTF monoclonal antibody ^32^, was applied to select specific human scFv against the peptide. Upon isolation of the scFv antibody against RTF from a large phage display library (RRID: AB_2636849) to evaluate the anti-proliferative and apoptosis effects of the anti-RTF scFv antibody, MTT and Annexin V assays were performed. The obtained results demonstrated a significant cell proliferation inhibition after 24 h treatment with 200–1000 scFv/cell for the three prostate cancer cell lines compared to untreated cells. A comparison among cell growth of three prostate cancer cell lines revealed that inhibition of cell growth in the PC-3 cell line was greater than two other cell lines (Du-145 and LNCaP). This might be due to a higher expression of RTF antigen in PC-3 cells and/or better accessibility of RTF to anti-RTF scFv antibody in PC-3 in comparison with Du-145 and LNCaP cell lines. Although Bermudez *et al.* ^17^ demonstrated that the amount of RTF mRNA in PC-3 is higher than in LNCaP cells, there has been no experiments to compare the levels of RTF mRNA in Du-145 cell line in comparison with PC-3 and LNCaP cell lines. Therefore, the higher growth inhibition in PC-3 after incubation with anti-RTF scFv could be due to higher amounts of RTF molecule in PC-3 than LNCaP.

No proliferation inhibition was detected for glioblastoma cell lines after incubation with different concentrations of the anti-RTF antibody compared with untreated cells, although the expression of RTF on glioblastoma cells has been confirmed ^33^. There could be several possible reasons for resistance of these cells to the anti-RTF effect. One could be the lack of RTF molecule accessibility to scFv antibody at the cell surface, due to antigen masking. The effect of masking of human epithermal growth factor receptor2 (ErbB2) via hyaluronan has previously been reported. The findings have demonstrated that masking of trastuzumab-binding epitope by hyaluronan took place in trastuzumab resistant breast cancer cell lines, such as JIMT-1. This masking contributes to the tumor cell escape from receptor-oriented therapy. Antigen masking can happen through overexpression of mucin (MUC) in tumor cells ^34^. In a study that was performed to understand the causative mechanism(s) of trastuzumab resistance in breast and some other cancers, it was discovered that MUC4 masks trastuzumab binding epitope of ErbB2, resulting in reduced binding of trastuzumab ^35^. Mishim *et al*. ^36^ demonstrated increased expression of podoplanin, which is a mucin-like transmembrane sialoglycoprotein in glioblastoma tumor cells. Therefore, a similar masking mechanism might also be attributed in glioblastoma cells, which precludes RTF binding to anti-RTF scFv antibody. Existence of other isoforms (a _1_, a _3_, and a _4_) of a subunit of proton pump on the cell surface can be considered as another possible mechanism that inhibits the anti-proliferative effects of anti-RTF scFv antibodies on U-87 MG and A-172 cell lines. In addition, the proton pump is not the only mechanism of pH regulation in tumor cells. A number of strategies are involved in control and regulation of pH in glioblastoma cell, such as sodium-proton exchanger-1 (NHE1). It has been demonstrated that U87 MG cell line increases the expression of NHE1 molecule in contrast to normal brain cells to maintain an optimal intracellular pH ^37^. However, the mechanism(s) involved in the non-responsiveness of U87 MG and A172 to anti-RTF scFv antibody remains to be elucidated.

It has been shown that proton pump inhibitors induce apoptosis in human B-cell tumors through a caspase-independent mechanism ^38^.The apoptosis-inducing effects of anti-RTF monoclonal antibody on ovarian carcinoma cells was assessed using Annexin V-FITC assay, and the J774A1 macrophage cell line incubated with anti-RTF showed a complete inhibition of surface ATPase activity ^39^ (US patent, US 7211257 B2). In addition, the role of the anti-RTF in T cell apoptosis has been shown ^18^. In the present study, the results of Annexin V-FITC assay were consistent with the MTT assay: apoptosis was induced in the three treated prostate cancer cells, however no evidence of apoptosis was observed in the treated glioblastoma cells. In recent years many efforts have been made to induce apoptosis in tumor cells through antibodies. For example, the anti-Fas monoclonal antibody was produced and exploited for apoptosis induction in several glioblastoma cell lines. Although some of glioblastoma cell line, such as LN-18 and LN-215, were sensitive to treatment with the monoclonal antibody against Fas, other cell lines, such as LN-308 and LN-405, showed resistance to anti-Fas antibody-mediated apoptosis. The reason for sensitivity was higher expression of Fas molecule in sensitive rather than in resistant cell lines ^40^. Single chain antibodies to some tumor markers, such as PSCA and IL25 receptor, have been capable of triggering apoptosis in tumor cells ^23, 41^. The lack of accessibility of RTF to scFv antibody and probably the presence of compensatory mechanism to pH regulation not only can inhibit an anti-proliferative effect, but also can protect the glioblastoma cells from undergoing apoptosis. By comparison, these characteristics were not observed for prostate cancer cells and the novel scFv selected in this study showed significant anti-cancer effects on the prostate cancer cells.

Due to several advantages of scFvs ^42^, a number of single chain antibodies have been selected against prostate cancer biomarkers, such as PSA, PSMA and PSCA ^41, 43, 44^. Although anti-PSMA scFv has shown promising effects for prostate cancer immunotherapy and has been introduced as a tool for building theranostic reagents for prostate cancer ^30^, it originated from a murine monoclonal antibody which induces human anti mouse antibody response (HAMA) ^45, 46^. Whereas the scFv selected in this study originated from human immunoglobulin genes and does not elicit any HAMA reaction. In addition, the ability for genetic manipulation can improve the antibody effect to produce fusion proteins with additional effector functions ^46– 49^. The inhibitory effect of human scFvs against prostate cancer was also reported by Vaday *et al.* ^50^. In that study, two scFvs were selected against CXCR4 and their inhibitory effects on CXCL12- mediated prostate cancer cell activation was investigated. The high affinity scFvs bound to receptor CXCR4 and inhibited its ligand, CXCL12, which resulted in cancer cell inhibition.

The panning process, as used by the present study, in the selection of scFvs against a target that enriches a phage antibody leads to isolation of specific antibodies with high affinity and high specificity. The novel anti-RTF single chain antibodies selected in this study with significant anti-proliferative and apoptotic functions on the three prostate cancer cell lines offers specific anti-prostate immunotherapy. Future efforts should be focused on testing the ability of anti-RTF scFv to inhibit prostate cancer growth in experimental models. Manipulation of the selected anti-RTF scFv and conjugation with a toxin may increase its ability to eliminate tumor cells and contribute to glioblastoma immunotherapy.

### Data availability

Dataset 1: Phage ELISA raw data. doi, 10.5256/f1000research.10803.d151807 ^51^

Dataset 2: Cell proliferation assay (MTT assay) raw data of three prostate cancer and two glioblastoma cell lines. doi, 10.5256/f1000research.10803.d151808 ^52^

Dataset 3: Apoptosis raw data for three prostate cancer and two glioblastoma cell lines. doi, 10.5256/f1000research.10803.d151809 ^53^
