# Supplementary material for: Cell growth inhibition and apoptotic effects of a specific anti-RTFscFv antibody on prostate cancer, but not glioblastoma, cells
Source: F1000Res. 2017 Feb 17;6:156. [Version 1] doi: 10.12688/f1000research.10803.1 (PMC5399964; doi:10.12688/f1000research.10803.1)
Supplement: Peer review history

# Peer review: Referee response for version 1

**DOI:** 10.5256/f1000research.11649.r20809

**Published:** 2017-04-18

**Authors:** Masoumeh Rajabibazl

**Affiliations:** 1 Department of Clinical Biochemistry, Faculty of Medicine, Shahid Beheshti University of Medical Sciences, Tehran, Iran

This is an interesting article and topic. The article is well written.

 Research to find new ways to treatment cancer, especially common cancers such as prostate, is valuable. Research on immunotherapy of various cancers with full length antibodies or better than them, antibody fragments such as scFv that have the ability to inhibit the growth of cancer cells is important.

 The study is well-designed and good & valid results have been achieved.

 Selection of anti-RTF scFv antibody with anti-proliferative and apoptosis effects against prostate cancer cells is promising. But since scFvs have disadvantages in comparison with whole mAb, in vivo study in future studies is recommended.

 It is necessary to impose the following points:

1. It is essential to the results of the phage ELISA be added.
2. Figures 1, 2 and 3 further explanation needed.

I have read this submission. I believe that I have an appropriate level of expertise to confirm that it is of an acceptable scientific standard.

# Peer review: Referee response for version 1

**DOI:** 10.5256/f1000research.11649.r21172

**Published:** 2017-03-29

**Authors:** Judith Niesen, Mira Woitok

**Affiliations:** 1 Fraunhofer Institute for Molecular Biology and Applied Ecology IME, Aachen, Germany

Nejatollahi et al. presented in their study a novel human scFv fragment against RTF. The manuscript is well written. The results are demonstrated in a detailed and informative way. The manuscript is only ready for indexing after considering the following major and minor comments:

**Abstract: **

- Beginning: Would be better to say scFvs instead of scFv. Use the plural from.
- Methods: library of scFvs instead of scFv; “The anti-proliferative and apoptotic effects of the antibody” please correct to scFv.
- Results: Please add: of the prostate CANCER cells after 24 h.
- Conclusions: please add: were greater than IN Du-145 and LNCaP cells.
- Please write Anti-RTFscFv consistent.

**Introduction**:

 You describe the properties of scFvs, but the property of human origin is nothing special for scFvs, MAbs could also be human and scFvs could also be of mouse origin. Of course it is and advantage if the scFv is human.

 The introduction is a bit short, compared to the abstract in length. Normally the abstract is much shorter than the introduction in most of the manuscripts. You could go a bit more in detail about the way of function of the scFvs in the cancer cells (or you can mention this point in the discussion section).

**Methods:**

 AnnexinV/FITC Assay please specify which well plate you use 6-well, 12-well, 24-well?

 In best case a negative control should also be included such as a non-binding scFv, to compare unspecific effects and/or a negative control cell line, not incubating the antigen/receptor etc.

 Make sure to write 1h (1 h, hrs) or 10 mg/ml (10 mg/ml) consistent throughout the manuscript.

 Please correct: “U-87 MGand A-17”.

 Please add the concentration unit (100, 200, 500, 1000 scFv/cell), e.g. nM.

 Cell proliferation assay: Do you used a positive control (100% killing) as blank value?

**Results:**

 Selection of an anti-RTF scFv antibody: please clarify which clone was used as tested scFv.

 Figure 1: Please relocate (B) in the figure legend.

 Figure 2: Please delete title X and Data 1.

 MTT Assay: It would be great to determine an EC/IC _50_ values for better comparison to similar acting scFvs/antibodies. Moreover, the unit of scFv/cell is not usual, in our opinion. Please write the concentration in e.g. molarity.

 AnnexinV/assay: It would be great if you could also obtain and show the dot blot of the histograms, which is the normal way to demonstrate apoptosis. Because in the dot blots you can compare early and late apoptotic/necrotic cells as well as live cells.

 It would be nice to have binding analysis of the scFv.

**Discussion:**

 Did you have any affinity data of your new scFvs? Could you give information about the way of function of the scFvs in the cancer cells. For mAbs it could be ADCC, CDC or blocking of signaling pathways. For scFv-based Immunotoxins its receptor mediated endocytosis and the toxin acts in the cancer cells…

 It would be nice to have a comparison between the toxicity of the full length mAb and the novel scFv. Are there any data available?

 Binding analysis of the novel scFv to the used cell lines would be advantageous for the discussion.

We have read this submission. We believe that we have an appropriate level of expertise to confirm that it is of an acceptable scientific standard, however we have significant reservations, as outlined above.

# Peer review: Referee response for version 1

**DOI:** 10.5256/f1000research.11649.r20321

**Published:** 2017-02-24

**Authors:** Issam Alshami

**Affiliations:** 1 College of Medicine, Taibah University, Medina, Saudi Arabia

In this study, the researchers discussed well the inhibitory effects of a specific anti-RTF scFv and compared between three types of prostate cancer and two types of glioblastoma cells. The results are interesting where it has been found that the selected anti-RTF scFv antibody could be an effective new alternative for prostate cancer immunotherapy. The present study provides scientific evidence regarding that.

 Data and references are update and sufficient information has been provided for replication of the experiment.

 The anti-RTFscFv which is selected in this research is a novel antibody. The anti proliferative and apoptotic effects reported here make this antibody an attractive agent for immunotherapy against prostate cancer and other cancers express this antigen. As the authors mentioned the unique properties of scFv antibodies have made these small libraries ideal antibodies for targeted therapy. The anti- RTF scFv which  blocks RTF will lead irregulation of  extracellular and intracellular pH in cells and would lead to cancer cell death as shown by the authors. The in vivo study using this antibody is recommended.

I have read this submission. I believe that I have an appropriate level of expertise to confirm that it is of an acceptable scientific standard.
